# Adrenal washout CT in patients with no history of cancer: a waste of time?

**DOI:** 10.1007/s00261-024-04333-5

**Published:** 2024-05-21

**Authors:** Timo van Aswegen, Ben Trinh, Angela Jacques, Glen Lo

**Affiliations:** 1 Sir Charles Gairdner Hospital, Nedlands, WA Australia; 2 Department of Research, Sir Charles Gairdner Hospital, Nedlands, WA Australia; 3 Institute for Health Research, The University of Notre Dame, Fremantle, WA Australia; 4 Curtin University, Bentley, WA Australia; 5 Diagnostic Imaging, Sir Charles Gairdner and Osborne Park Hospital Care Group, Hospital Avenue, Nedlands, WA Australia

**Keywords:** Adrenal, Nodule, Washout, Phaeochromocytoma, Adenoma

## Abstract

**Purpose:**

To validate the diagnostic performance of adrenal washout CT in patients *without known malignancy* in a Western Australian population.

**Methods:**

A radiology information system (RIS) search for CT reports containing “adrenal” and “washout” across six networked metropolitan public hospitals between January 2005 and November 2021. Homogenous nodules ≥ 1 cm, ≥ 10 HU without a suspected functional component in patients without a history of malignancy were included. Reported absolute and relative washout percentages were recorded and re-measured from unenhanced, 60-s portal venous and 15-min delayed phase imaging and compared to either histopathological or CT follow up for growth (≥ 12 months) reference standards.

**Results:**

2653 studies were screened with 191 meeting inclusion criteria. 105 nodules underwent washout CT and then had either histopathological (12 patients) or CT follow up (93 patients) reference standards available.

Reported absolute washout (aWO) estimated sensitivity and specificity for malignant/indeterminate nodules was low at 33% (95% CI 25–43%) and 77% (95% CI 68–84%) respectively. Reported relative washout (rWO) sensitivity and specificity were 56% (95% CI 46–65%) and 69% (95% CI 60–77%) respectively. Negative predictive values for both aWO and rWO were reassuring at 92% (95% CI 86–96%) and 94% (95%CI 88–97%).

**Conclusion:**

Our study validates a recent report suggesting that adrenal washout has poor sensitivity for and consequent limited utility to exclude malignancy in patients with no cancer history. However, patients with incidental adrenal nodules < 4 cm in size with benign washout can be reassured by the high negative predictive value and worked up to exclude functional adenoma and re-imaged in a year to confirm no growth.

**Graphical Abstract:**

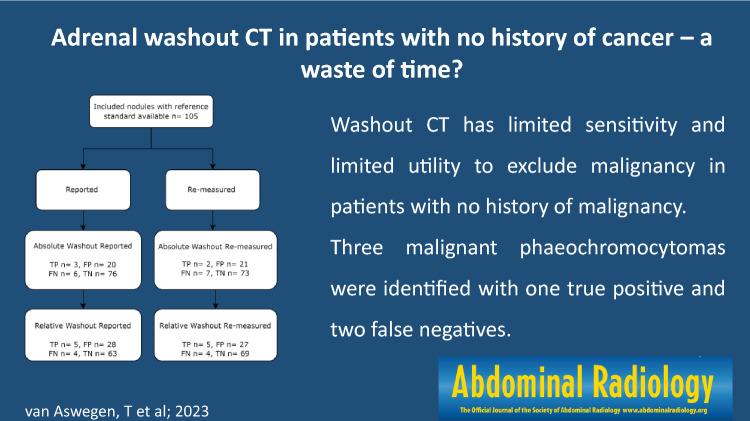

## Introduction

Adrenal incidentalomas are found in approximately 5% of patients undergoing CT (computed tomography) imaging and often bear a management problem [1]. In patients with no cancer history, adrenal incidentalomas are almost always benign [2]. However, CT-based diagnostic characterization of adrenal incidentalomas is not always possible, with benign and malignant appearances overlapping [2]. As a result, there is potential for over-diagnosis of malignancy with unnecessary biopsies being required to provide clinicians and patients with diagnostic certainty.

Adrenal incidentalomas are nodules measuring greater than 1 cm in diameter and can be benign with no follow up required, or malignant (primary or secondary) with further evaluation required. In addition, adrenal incidentalomas can also be functional or non-functional [2]. Due to widespread access to cross sectional imaging, many patients with an adrenal incidentaloma will undergo further characterization with CT imaging at diagnosis. Lesion characterization is multifactorial and considers size, margin, heterogeneity, intracellular lipid content, macroscopic fat content and enhancement [3].

Previous studies have shown that an adrenal nodule with an unenhanced density less than 10 HU (lipid-rich), is highly specific for an adrenal adenoma. These lipid-rich adrenal adenomas comprise up to 70% of adrenal adenomas [2, 4, 5]. Adrenal adenomas with an unenhanced density of more than 10 HU are lipid-poor and indeterminate on single phase imaging. These nodules require adrenal washout imaging to differentiate them from other adrenal tumours which can have similar unenhanced imaging characteristics, such as metastases or primary adrenal tumours such as pheochromocytoma and adrenal cortical carcinoma. Adrenal washout CT involves performing unenhanced, portal venous and ideally 15-min enhanced delayed phase imaging with individual density measurements being used to calculate absolute and relative washout [6]. Nodules with absolute washout of 60% or greater are considered benign whilst absolute washout of less than 60% is considered indeterminate/ malignant. When unenhanced imaging is not available, a relative washout of ≥ 40% is suggestive of a benign nodule. Previous studies have shown that washout CT has high sensitivity and specificity for adrenal adenomas [7–9]. For example, one study reported absolute washout of at least 60% to have a sensitivity of up to 98% and specificity of up to 92% for differentiating lipid-poor adenomas and non-adenomas [9]. Unfortunately, most prior studies were performed in non-incidental populations i.e., patients with known malignancy.

Unfortunately, there is considerable overlap of washout characteristics between lipid poor adenomas and phaeochromocytomas, with previous studies reporting up to one third of phaeochromocytomas demonstrating the same washout pattern as adenomas [10–13]. As phaeochromocytomas cannot be accurately differentiated from adenomas with washout CT, biochemical testing is often required to confirm the diagnosis of phaeochromocytoma, more specifically, 24-h urine metanephrines are most useful, with reported sensitivity and specificity of more than 90% [14]. Despite initial concerns regarding the potential of inducing a hypertensive crisis, administration of non-ionic contrast in the setting of suspected phaeochromocytoma is considered safe and CT is widely used in the work up of phaeochromocytoma [15].

Recommendations for the management of adrenal incidentalomas were published by the American College of Radiology (ACR) in 2010 with nodules between 1 and 4 cm and with mean density of ≤ 10 HU being essentially diagnostic of a benign lesion [16]. In the absence of these features, stability in nodule size over at least 1 year is also indicative of a benign nodule, particularly in patients with no cancer history. These guidelines were updated in 2017, with a salient change including the further work up of masses more than 2 cm in size due to the increased prevalence of malignancy among larger lesions. The 2017 ACR guideline also suggests that nodules between 2 and 4 cm in size with benign features on washout CT (absolute washout of > 60% and relative washout of > 40%) are likely benign adenomas and do not require follow up. Follow up CT imaging is, however, recommended at 6 to 12 months for nodules that are indeterminate based on washout imaging to assess for stability [17].

Variation exists among other published guidelines from the 2002 National Institutes of Health (NIH), 2009 American Association of Clinical Endocrinologists (AACE) and American Association of Endocrine Surgeons (AAES) and 2007 New England Journal of Medicine (NEJM) Clinical Practice Guidelines. The NIH, NEJM and AACE/AAES guidelines differ from the current ACR guidelines in that they recommend all nodules that are less than 4 cm in size require imaging follow up at varying intervals between 3 and 24 months after the initial diagnosis [18–20]. Biochemical testing has previously been recommended only in patients with clinical symptoms and signs of adrenal hyperfunction [21]. Current guidelines recommend biochemical evaluation in all patients with adrenal incidentalomas [17–20]. This comprises of a dexamethasone suppression test as well as plasma and/ or urine metanephrines and catecholamines with serum aldosterone and/or renin levels also recommended, depending on the presence of hypertension. In addition, recent advances in nuclear medicine imaging technology such as positron electron tomography (PET) should also be considered in the evaluation of indeterminate and enlarging nodules, particularly in patients with known malignancy [22, 23].

A recently published study performed by Corwin et al. [24] has called into question the diagnostic performance of washout CT in patients without known malignancy. It concluded that washout of at least 60% had suboptimal sensitivity of 75% and specificity of 71% across all nodules, including phaeochromocytoma. The aim of this study is to validate these findings within the Western Australian population as our public hospitals share the same PACS and service the majority of the state’s population.

## Methods

This was a retrospective cross-sectional study. A radiology information system (RIS) search was conducted to identify all abdominal CT examinations performed between 1st January 2005 and 12th November 2021 with radiology reports containing the terms “adrenal” and “washout”. Data were collected across six public hospitals in Perth, Western Australia (Sir Charles Gairdner Hospital, Fiona Stanley Hospital, Royal Perth Hospital, Osborne Park Hospital, Fremantle Hospital and Rockingham Hospital).

### Inclusion and exclusion criteria

The inclusion criteria consisted of homogenous adrenal nodules of ≥ 1 cm, with a mean density of ≥ 10 HU and without a suspected functional component in patients of at least 18 years of age without a history of malignancy. Although nodules > 4 cm are recommended for resection in the ACR White Paper management guideline we included these lesions that had undergone adrenal washout CT as a subgroup, as there was follow up data available [17].

Patients were excluded if the CT examination request form or digital medical record noted a history of malignancy or functional component suspected to be related to an adrenal nodule. Nodules were also excluded based on size (smaller than 1 cm) or if nodules demonstrated features of benignity such as macroscopic fat or demonstrated suspicious features such as heterogeneity, irregular margins, and vascular invasion. Nodules confirmed as an adrenal adenoma on magnetic resonance imaging (MRI) were also excluded, as no CT characterization was done.

### Imaging technique

A dedicated CT imaging protocol for the work up of adrenal lesions consisted of acquisitions obtained at 120 kVp, variable mAs and with slice thicknesses ranging between 1 and 5 mm. Each study consisted of three phases; unenhanced, portal venous (acquired at 60–80 s after contrast administration) and delayed phase imaging (acquired at 10–15 min). Each examination was analyzed using Agfa IMPAX software (version 6.7.0.6011) on radiology workstations.

### Image evaluation

**Fig. 1:**
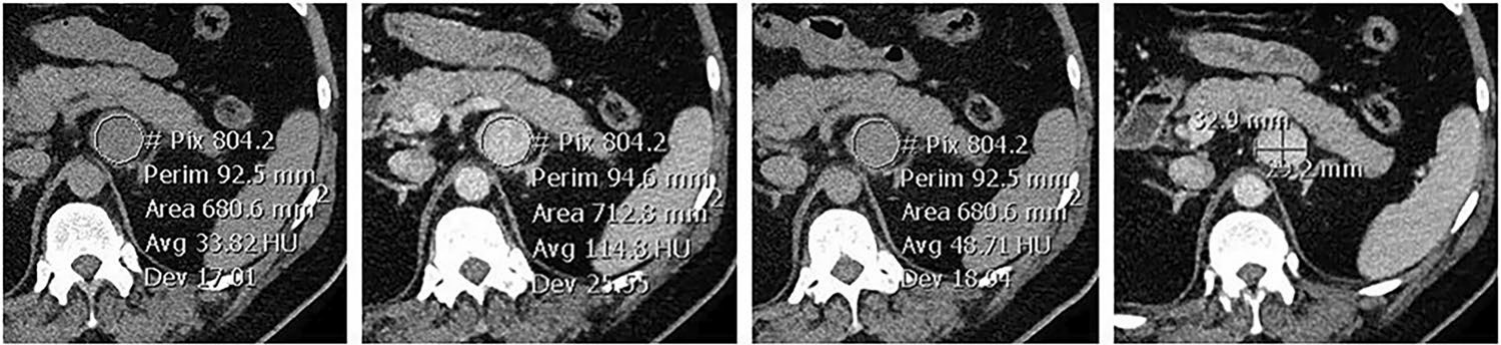
Adrenal washout computed tomography with axial slices demonstrating a left adrenal nodule with mean attenuation measurements on non-contrast enhanced (left), portal venous (middle left) and delayed (middle right) acquisitions. Long and short-axis measurements (right)

Nodule size, appearances and characteristics were obtained both from the radiology report and repeated measurements performed by the primary author. Nodule size was obtained using the long-axis diameter. To acquire nodule attenuation for each phase of the washout protocol, a circular region of interest (ROI) marker was placed over the adrenal nodule, with coverage of at least two thirds of the diameter, as shown in Fig. 1. Effects of partial volume averaging were minimized by ensuring that the peripheral margin of the nodule was not included in the ROI and that the nodule was visible on contiguous slices above and below the ROI. The brand of iodinated contrast material varied depending on the institution (Omnipaque 350 being the most commonly used agent), with volumes used ranging between 75 and 120 mL.

### Washout calculation

Using nodule attenuation measurements, absolute and relative washout values were calculated. The formula used to calculate absolute washout was [(portal venous phase HU)—(delayed phase HU)]/[(portal venous phase HU)—(unenhanced HU)] × 100. Relative washout was calculated using [(portal venous phase HU)—(delayed phase HU)] / (portal venous phase HU) × 100.

Reported absolute and relative washout percentages were recorded and re-measured from unenhanced, 60-s portal venous and 15-min delayed phase imaging. For a nodule to be characterized as benign based on washout, cut offs of ≥ 60% absolute and ≥ 40% relative were used and compared with histopathological or CT growth follow up reference standards.

### Reference standards

Reported and re-measured absolute and relative washout percentages were compared with either histopathological and/or CT growth reference standards. Histopathological reference data were obtained from the digital medical record (DMR) following biopsy or surgical resection of an adrenal nodule. Each pathology report of a confirmed phaeochromocytoma also provided the Phaeochromocytoma of the Adrenal gland Scaled Score (PASS), a score based on the presence or absence of 12 histological features to improve the distinction between benign and malignant nodules for the purposes of prognostication. A score of < 4/20 was used to assign probable benignity and a score of ≥ 4/20 defines a nodule with malignant potential [25].

In the absence of a histopathological result, A CT-growth reference standard was used. This involved assessment of nodule growth on CT, MRI or PET-CT studies which demonstrate the index nodule in its entirety and were performed at least 12 months before or 12 months after the washout CT study. The earliest prior study and/ or the most recent subsequent study were performed when multiple studies were available for comparison. Nodules were classified as benign if there was no growth or growth of ≤ 3 mm in long-axis diameter. Nodules were classified as malignant if growth of ≥ 8 mm in long axis diameter was demonstrated. Nodules were classified as indeterminate if growth between 3 and 8 mm was demonstrated.

### Statistics

Demographic data were summarized using descriptive statistics. Diagnostic accuracy (sensitivity, specificity, negative and positive predictive values) and prevalence were calculated using 2 × 2 tables. Values were calculated for nodules of all size, as well as nodules < 4 cm and ≥ 4 cm. 95% confidence intervals are reported. Interobserver reliability was calculated and reported using Cohen’s Kappa and statistically significant results were assigned a *p* value of < 0.05.

## Results

**Fig. 2:**
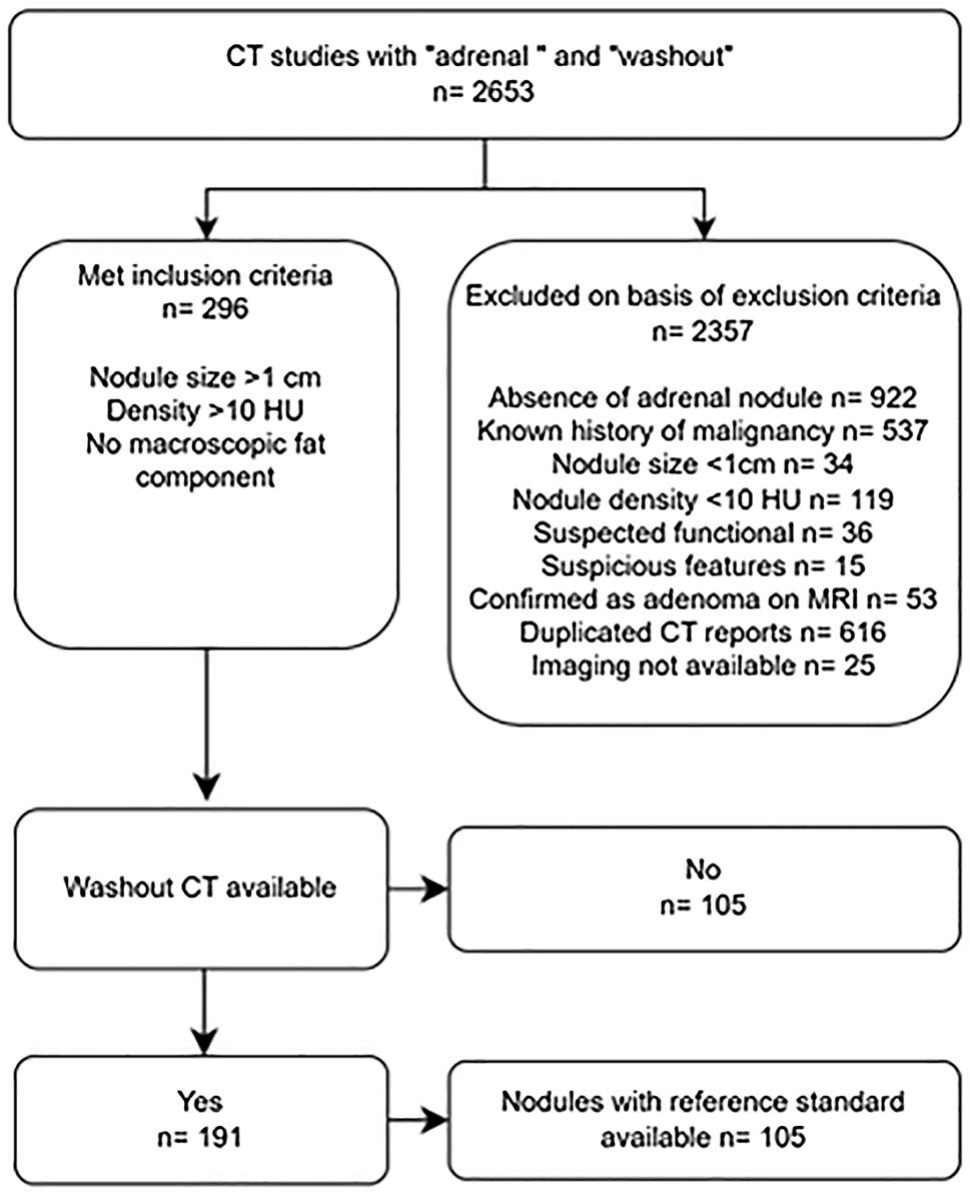
Flowchart of included and excluded patients

As shown in Fig. 2, a total of 2653 CT reports were reviewed. A total of 296 met the initial inclusion criteria. Of these, 191 underwent an adrenal washout CT and 105 had a nodule with either a histopathological or CT growth reference standard available. Reasons for exclusion of a large number of nodules are also shown in Fig. 2. Median subject age was 55 (range 28–82) years. Distribution of sex was equitable with 55 female and 50 male participants.

In this study, the disease entity was defined as indeterminate/ malignant nodules. The estimated prevalence of disease across nodules of all size was 9.5% (10/105) when including all indeterminate/ malignant nodules. The prevalence of malignancy alone was lower at 4.8% (5/105). The prevalence of malignancy in nodules under 4 cm is 3% (3/100), or 8% (8/100) when indeterminate/ malignant nodules are grouped. Prevalence of malignancy in nodules 4 cm or larger was 40% (2/5).

Of the 105 nodules with a reference standard available, 12 were assessed against a histopathological reference standard, and 93 were assessed against the CT growth interval reference standard. A total of five nodules were classified as malignant, four based on histopathology and one on the basis of CT growth criteria. Five nodules were indeterminate based on CT growth criteria and did not have a histopathological diagnosis for definitive classification. A total of 95 nodules were classified as benign based on histopathology (10 nodules) and CT growth criteria (85 nodules).

**Fig. 3:**
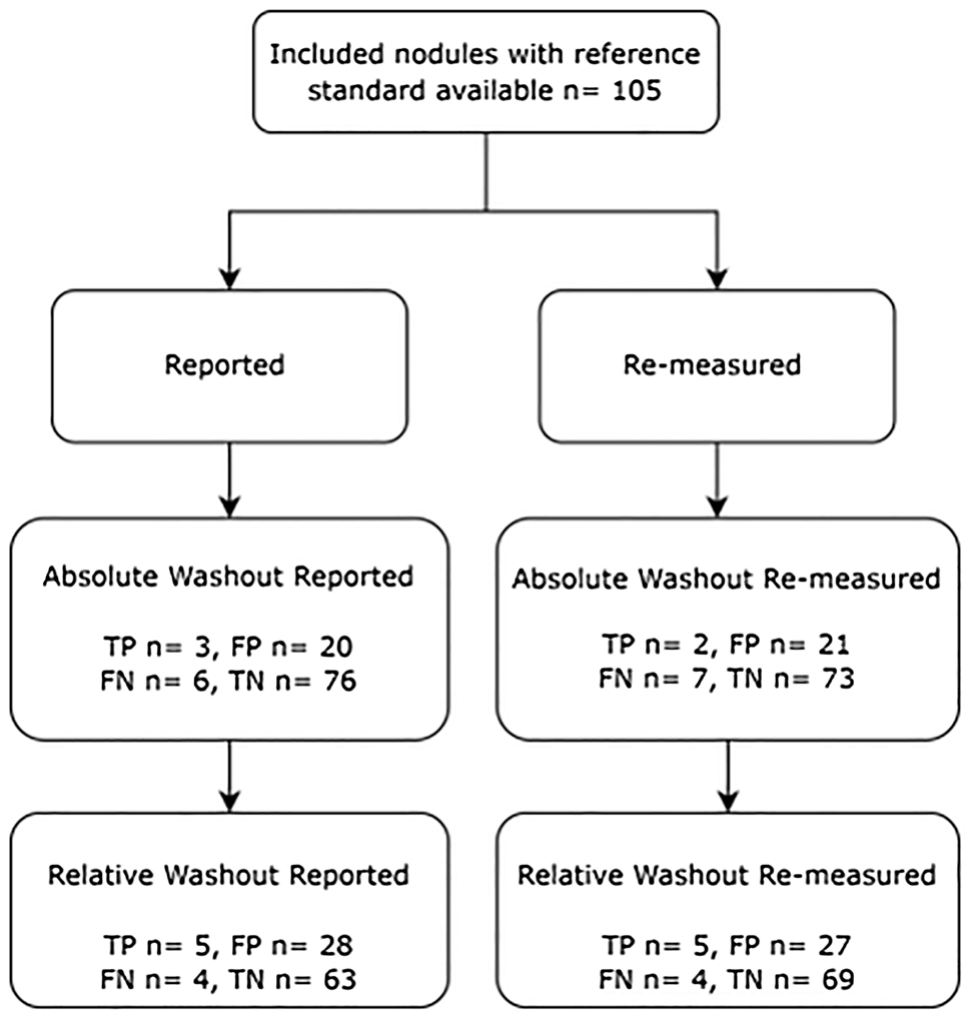
Flow chart summarizing the raw diagnostic accuracy of absolute and relative washout reported and re-measured nodules. *TP* true positive, *FP* false positive, *FN* false negative and *TN* true negative

Raw diagnostic accuracy values between the reported and remeasured absolute and relative washout across all nodules are highlighted in Fig. 3. The sensitivity of reported absolute washout was 33% (95% CI 25–43%) and the specificity was 77% (95% CI 68–84%). The negative predictive value (NPV) was 92% (95% CI 86–96%) and the positive predictive value (PPV) was 12% (95% CI 7–20%). The sensitivity of reported relative washout was 56% (95% CI 46–65%) and specificity 69% (95% CI 60–77%). The NPV was 94% (95% CI 88–97%) and the PPV was 15% (95% CI 10–23%). The diagnostic performance for reported relative washout could not be assessed for five nodules due to nodule densities and relative washout values not being conveyed in the study report.

Nodules were also grouped by size (< 4 cm and ≥ 4 cm), as shown in Table 1. For nodules < 4 cm, the reported absolute washout sensitivity was 29% (95% CI 4–71%) and specificity 76% (95% CI 66–84%). Reported relative washout sensitivity was 56% (95% CI 21–86%) and specificity 57% (95% CI 43–69%). The negative predictive values for absolute and relative washout were 93% (95% CI 90–96%) and 89% (95% CI 80–95%).

**Table 1:** Diagnostic accuracy of reported absolute washout stratified by nodule size

Nodule size	Diagnostic classification	Absolute washout reported	Relative washout reported
< 4 cm	Indeterminate/malignant *n* = 8 Benign *n* = 92	Sensitivity 29% (95% CI 4–71%) Specificity 76% (95% CI 66–84%) PPV 8% (95% CI 3–24%) NPV 93% (95% CI 90–96%)	Sensitivity 56% (95% CI 21–86%) Specificity 57% (95% CI 43–69%) PPV 16% (95% CI 9–27%) NPV 89% (95% CI 80–95%)
≥ 4 cm	Indeterminate/malignant *n* = 2 Benign *n* = 3	Sensitivity 50% (95% CI 1–99%) Specificity 9% (95% CI 9–99%) PPV 50% (95% CI 11–89%) NPV 67% (95% CI 29–91%)	Sensitivity 100% (95% CI 16–100%) Specificity 33% (95% CI 1–91%) PPV 50% (95% CI 31–69%) NPV 100%

For nodules ≥ 4 cm, reported absolute washout sensitivity was 50% (95%CI 1–99%) and specificity 9% (95% CI 9–99%). Reported relative washout sensitivity was 100% (95% CI 16–100%) and specificity 33% (95%CI 31–69%). The negative predictive values for absolute and relative washout were 67% (95% CI 29–91%) and 100%.

Results were internally validated by investigators re-measuring nodule size and re-calculating absolute and relative washout. Re-measured absolute washout values had good agreement with reported values (Kappa 0.697, *p* value < 0.001), as did relative washout (Kappa 0.838, *p* value < 0.001). Re-measured sensitivity and specificity for absolute washout were 22% (95% CI 15–31%) and 78% (95% CI 69–85%) respectively. Re-measured sensitivity and specificity for relative washout were 56% (95% CI 46–65%) and 72% (95% CI 63–80%) respectively. NPV was identical to reported values at 91% (95% CI 84–95%) and 95% (95% CI 88–98%) for re-measured absolute and relative washout respectively.

Table 2 summarizes the diagnoses made by histopathology. Four malignant nodules had a histopathological diagnosis, comprising of three malignant phaeochromocytomas and one adrenocortical carcinoma.

**Table 2:** Summary of diagnoses made by histopathology

Histopathological diagnosis	Number of nodules
Phaeochromocytoma (malignant)^a^	3
Phaeochromocytoma (benign)^a^	2
Adrenal ganglioneuroma	2
Adrenocortical adenoma	1
Adrenocortical carcinoma	1
Benign epithelial cyst (> 4 cm)	1
Benign not specified	1
Myelolipoma	1
Schwannoma (benign)	1

^a^Denotes classification of malignant potential as guided by the PASS (Phaeochromocytoma of the Adrenal gland Scaled Score)

A total of five nodules were histopathologically defined as phaeochromocytoma, three of which were classified as malignant and two as benign. The average size was 29 mm (range 17–50 mm). Mean size of the three malignant phaeochromocytomas was 29 mm (range 17–52 mm). These were of similar NECT density, ranging between 37 and 46 HU and demonstrated portal venous enhancement ranging between 61 and 81 HU. Reported absolute washout for the malignant phaeochromocytomas were − 13%, 71% and 89%. Only one malignant nodule had a follow up CT at 12 months, which interestingly did not demonstrate any growth. Both benign phaeochromocytomas demonstrated hypervascular enhancement (130 and 117 HU). Five nodules were 4 cm or greater in size with a mean size of 6.3 cm (range 4–14 cm). Of these, two were malignant (one adrenocortical carcinoma (14 cm) and one phaeochromocytoma (5 cm)) and three were benign (one benign epithelial cyst, two with no pathological diagnosis).

## Discussion

This study found a low prevalence of malignancy among homogenous adrenal incidentalomas size 1–4 cm in patients without known malignancy. Expectedly, prevalence of non-benignity was higher when indeterminate/ malignant nodules were grouped, particularly among nodules less than 4 cm in size. Washout CT has low sensitivity and specificity for the detection of indeterminate/ malignant nodules. When stratified for nodule size, sensitivity and specificity of washout CT remained low, with sensitivity and specificity of absolute washout reducing slightly for nodules below 4 cm with a similar NPV. Diagnostic performance remained suboptimal for nodules 4 cm or above, sensitivity of washout improved slightly to 50% however specificity was low.

The prevalence of indeterminate/malignant nodules was relatively high in this study, reported as 9.5% (10/105). This is higher than previous studies, almost certainly due to the inclusion of indeterminate nodules in this group. The prevalence is more comparable with prior studies when reporting malignant nodules only (including phaeochromocytomas), at 4.8%. For example, Corwin et al. [24] reported a prevalence of 4.2% across nodules of all size, 3.2% in nodules under 4 cm and 21% in those 4 cm or larger. The prevalence of malignancy in nodules under 4 cm in size is similar in the current study (3%). The prevalence of malignancy in nodules over 4 cm is higher, found to be as high as 40% in this study.

This study differs from previous studies in that adrenal adenomas have principally been defined as the ‘disease’, i.e., washout CT has been used to differentiate adrenal adenomas from non-adenomas. As such, the study performed by Corwin et al. [24] reported washout of 60% or more had a sensitivity of 67.6% and specificity of 77.8% for differentiating adenomas from non-adenomas (including phaeochromocytoma, as in this study). Given this study defined indeterminate/ malignant nodules as the ‘disease’ entity, the specificity is therefore comparable at 79%, as is the NPV, with the prior study reporting an NPV of 98% vs 93% in this study. Similarly, Akbulut et al. [26] also assessed the utility of washout in true adrenal incidentalomas and reported that absolute washout of 60% or more had a sensitivity of 67.6% and specificity of 77.8% for differentiation adenomas from non-adenomas, including phaeochromocytomas.

The sensitivity for the current study is significantly lower than both previously mentioned studies at 33%, presumably due to the combination of significant overlap in washout characteristics between indeterminate/ malignant nodules and benign nodules and a smaller sample size (336 vs 175 vs 105 nodules respectively). Other studies have also demonstrated significantly better diagnostic accuracy of washout CT, up to 98% sensitivity and 92% specificity, although these studies involved patients with known malignancy [7–9].

**Fig. 4:**
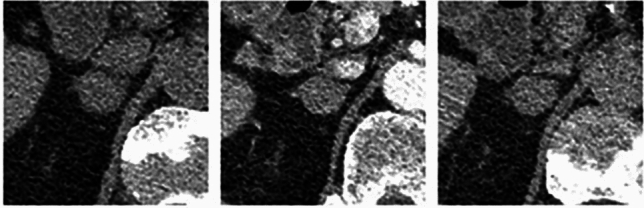
Non-enhancing malignant phaeochromocytoma demonstrated on axial CT acquisitions; left—non-contrast phase, 530 middle—portal venous phase and right—delayed phase

**Fig. 5:**
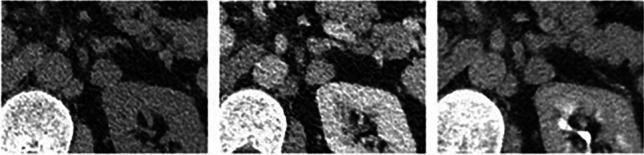
Axial CT images of a left adrenal nodule with benign washout characteristics, later proven to be malignant phaeochromocytoma following resection; left—non-contrast phase, middle—portal venous phase and right—delayed

The prevalence of phaeochromocytoma across all adrenal incidentalomas has previously been reported to be as high as 14% [27]. Prevalence was lower in the current study, reported as 4.8% (5/105). Two phaeochromocytomas (40%, 2/5) demonstrated hypervascularity on washout imaging, which were classified as benign on pathology. In the study performed by Corwin et al. [24], one third (33.3%) of phaeochromocytomas demonstrated absolute washout of 60% or more. Although the two hypervascular nodules identified in this study were classified as benign on histopathology, these remain likely to have functional implications. In addition, it is well established that washout CT does not distinguish hypervascular phaeochromocytoma from hypervascular metastases such as renal cell carcinoma or hepatocellular carcinoma [28]. Three phaeochromocytomas were classified as malignant on histopathology using the PASS tool [25]. As shown in Fig. 4, one malignant phaeochromocytoma measured 20 mm in size, had absolute washout of -13% and relative washout of -3%, indicative of no washout, possibly due to intra-lesional hemorrhage or degeneration, although degeneration is considered uncommon in small phaeochromocytomas [28]. A systematic review conducted in 2012 found that up 50% of hemorrhagic adrenal masses were phaeochromocytomas, likely due to their high vascularity leading to rapid growth and increased intra-capsular pressure, predisposing to capsular rupture and hemorrhage [29]. As well as being seen in the context of trauma, adrenal hemorrhage could also be the initial stigmata of an underlying adrenal mass lesion, re-iterating the importance of imaging to assess for resolution over the expected clinical course [30]. The remaining two malignant phaeochromocytomas were mischaracterized by washout (two false negatives). Of these two, one was large (52 mm) with an absolute washout of 71%. As demonstrated in Fig. 5, the third had the most benign imaging characteristics i.e., it was small (17 mm) and had an absolute washout of 89%. These data support the previously demonstrated overlap of absolute and relative washout values between adrenal adenoma and phaeochromocytoma, particularly in smaller nodules which are less likely to contain regions of hemorrhage, necrosis or calcification [31]. This reiterates the importance of biochemical testing as an adjunct to washout CT.

Multiple guidelines have been proposed for the management of adrenal incidentalomas [17–20]. The ACR white paper guidelines updated in 2017 informs the management of adrenal incidentalomas across the institutions involved in this study. This guideline suggests that in patients with no history of malignancy, incidental nodules between 1 and 2 cm in size are likely benign and 12 month follow up should be considered (in the absence of prior imaging). Nodules between 2 and 4 cm should undergo a washout CT with absolute washout of 60% or more or relative washout of 40% or more being suggestive of a benign adenoma, with no follow up imaging being recommended. The current study supports the recommendation that nodules with indeterminate washout features be followed up, noting that although the prevalence of malignancy is low among nodules < 4 cm in size, a significant subset could not be characterized as either benign or malignant. Further, we also suggest that the sensitivity and specificity of CT washout is suboptimal to inform the need for further work up and that follow up CT without washout is likely to identify growing nodules that require further biopsy or excision, with a lower radiation dose.

The 2017 ACR guideline also supports a size threshold where the risk of malignancy increases significantly. As such, PET imaging, biopsy or resection are recommended for nodules of 4 cm or greater. As with Corwin et al. [24], this study supports such a size threshold given the increased prevalence of malignancy (up to 40% in this study). However, due to the inherent low prevalence of large homogenous nodules, only a small number of nodules met the inclusion criteria, limiting statistical power.

This study has several limitations. Firstly, it was retrospective in nature. Secondly, CT growth follow up made up the reference standard for most nodules with only a small proportion having a histopathological reference standard (93 vs 12 respectively). This is predominantly due to the very small number of nodules with benign imaging appearances requiring excision or biopsy, particularly if they are under 4 cm in size. As a result, the results of this study can only be generalized to nodules that meet the inclusion criteria. Further, growth rates were based on the same thresholds used by Corwin et al. [24], noting there is no accepted gold standard growth rate that predicts malignancy in adrenal nodules [32, 33]. Thresholds are fundamentally designed to maximize specificity for both benignancy and malignancy. Thirdly, the process of including and excluding nodules will contain inherent subjectivity. For example, nodule eligibility was predominantly based upon the description of nodules in radiology reports which includes subjective assessment of suspicious features such as heterogeneity. As such, the single adrenocortical carcinoma included in this study may be felt to be heterogenous depending on the level of experience of an investigator. We attempted to minimize this by investigators assessing for suspicious features at the time of repeating measurements for each nodule that met inclusion criteria, with nodules with borderline characteristics being assessed by the most senior investigator (abdominal fellowship subspecialist radiologist with 10 years’ post fellowship experience) for eligibility. We also reviewed the clinical history of the study for the purpose of excluding studies noting a previous history of malignancy or those with a suspected functional nodule, however no consideration was made as to whether a symptomatic nodule (i.e., causing pain) was the indication for the CT study. Despite assessing CT studies performed across six institutions over 15 years, the sample size was smaller than previous studies, likely exacerbated by the inherent low prevalence of adrenal malignancy which expectedly becomes less prevalent in patients with no history of other malignancy. Further, the results of this study can only be applied to incidentally detected nodules in patients with no history of malignancy. The assessment of nodules in patients with known malignancy would necessitate additional work up such as PET imaging or biopsy ± excision, particularly in those with a potentially hypervascular primary malignancy.

## Conclusion

Our study validates the recent study performed by Corwin et al., suggesting that although the prevalence of malignancy is low among truly incidental nodules between 1 and 4 cm in size, washout CT has limited sensitivity and limited utility to exclude malignancy. Reassuringly, these data reassure that washout CT has a high negative predictive value for malignancy in patients without known malignancy, so after exclusion of a functional tumor, it is appropriate for an adrenal mass under 4 cm in size with benign washout to be followed up in one year to assess for interval growth. The data also suggests that in this population, indeterminate nodules under 4 cm in size may also be followed up with a single phase or non-contrast study, providing a functional component has been excluded.

## References

[1] Boland, G. W., Lee, M. J., Gazelle, G. S., Halpern, E. F., McNicholas, M. M., & Mueller, P. R. (1998). Characterization of adrenal masses using unenhanced CT: an analysis of the CT literature. *AJR. American journal of roentgenology*, *171*(1), 201–204. 10.2214/ajr.171.1.9648789. 9648789 10.2214/ajr.171.1.9648789

[2] Boland, G. W., Blake, M. A., Hahn, P. F., & Mayo-Smith, W. W. (2008). Incidental adrenal lesions: principles, techniques, and algorithms for imaging characterization. *Radiology*, *249*(3), 756–775. 10.1148/radiol.2493070976. 19011181 10.1148/radiol.2493070976

[3] Pokharel, S. S., Macura, K. J., Kamel, I. R., & Zaheer, A. (2013). Current MR imaging lipid detection techniques for diagnosis of lesions in the abdomen and pelvis. *Radiographics : a review publication of the Radiological Society of North America, Inc*, *33*(3), 681–702. 10.1148/rg.333125068. 23674769 10.1148/rg.333125068

[4] Lee, M. J., Hahn, P. F., Papanicolaou, N., Egglin, T. K., Saini, S., Mueller, P. R., & Simeone, J. F. (1991). Benign and malignant adrenal masses: CT distinction with attenuation coefficients, size, and observer analysis. *Radiology*, *179*(2), 415–418. 10.1148/radiology.179.2.2014283. 2014283 10.1148/radiology.179.2.2014283

[5] Korobkin, M., Brodeur, F. J., Yutzy, G. G., Francis, I. R., Quint, L. E., Dunnick, N. R., & Kazerooni, E. A. (1996). Differentiation of adrenal adenomas from non adenomas using CT attenuation values. *AJR. American journal of roentgenology*, *166*(3), 531–536. 10.2214/ajr.166.3.8623622. 8623622 10.2214/ajr.166.3.8623622

[6] Korobkin, M., Brodeur, F. J., Francis, I. R., Quint, L. E., Dunnick, N. R., & Londy, F. (1998). CT time-attenuation washout curves of adrenal adenomas and non adenomas. *AJR. American journal of roentgenology*, *170*(3), 747–752. 10.2214/ajr.170.3.9490968. 9490968 10.2214/ajr.170.3.9490968

[7] Caoili, E. M., Korobkin, M., Francis, I. R., Cohan, R. H., & Dunnick, N. R. (2000). Delayed enhanced CT of lipid-poor adrenal adenomas. *AJR. American journal of roentgenology*, *175*(5), 1411–1415. 10.2214/ajr.175.5.1751411. 11044054 10.2214/ajr.175.5.1751411

[8] Peña, C. S., Boland, G. W., Hahn, P. F., Lee, M. J., & Mueller, P. R. (2000). Characterization of indeterminate (lipid-poor) adrenal masses: use of washout characteristics at contrast-enhanced CT. *Radiology*, *217*(3), 798–802. 10.1148/radiology.217.3.r00dc29798. 11110946 10.1148/radiology.217.3.r00dc29798

[9] Caoili, E. M., Korobkin, M., Francis, I. R., Cohan, R. H., Platt, J. F., Dunnick, N. R., & Raghupathi, K. I. (2002). Adrenal masses: characterization with combined unenhanced and delayed enhanced CT. *Radiology*, *222*(3), 629–633. 10.1148/radiol.2223010766. 11867777 10.1148/radiol.2223010766

[10] Patel, J., Davenport, M. S., Cohan, R. H., & Caoili, E. M. (2013). Can established CT attenuation and washout criteria for adrenal adenoma accurately exclude pheochromocytoma?. *AJR. American journal of roentgenology*, *201*(1), 122–127. 10.2214/AJR.12.9620. 23789665 10.2214/AJR.12.9620

[11] Park, B. K., Kim, B., Ko, K., Jeong, S. Y., & Kwon, G. Y. (2006). Adrenal masses falsely diagnosed as adenomas on unenhanced and delayed contrast-enhanced computed tomography: pathological correlation. *European radiology*, *16*(3), 642–647. 10.1007/s00330-005-0017-0. 16215735 10.1007/s00330-005-0017-0

[12] Park, B. K., Kim, C. K., Kwon, G. Y., & Kim, J. H. (2007). Re-evaluation of pheochromocytomas on delayed contrast-enhanced CT: washout enhancement and other imaging features. *European radiology*, *17*(11), 2804–2809. 10.1007/s00330-007-0695-x. 17549484 10.1007/s00330-007-0695-x

[13] Schieda, N., Alrashed, A., Flood, T. A., Samji, K., Shabana, W., & McInnes, M. D. (2016). Comparison of Quantitative MRI and CT Washout Analysis for Differentiation of Adrenal Pheochromocytoma From Adrenal Adenoma. *AJR. American journal of roentgenology*, *206*(6), 1141–1148. 10.2214/AJR.15.15318. 27011100 10.2214/AJR.15.15318

[14] Guller U, Turek J, Eubanks S, Delong ER, Oertli D, Feldman JM. (2006). Detecting Pheochromocytoma. Annals of Surgery, *243*(1), 102–7. 10.1097/01.sla.0000193833.51108.24. 16371743 10.1097/01.sla.0000193833.51108.24 PMC1449983

[15] Bessell-Browne, R., & O'Malley, M. E. (2007). CT of pheochromocytoma and paraganglioma: risk of adverse events with i.v. administration of nonionic contrast material. *AJR. American journal of roentgenology*, *188*(4), 970–974. 10.2214/AJR.06.0827. 17377032 10.2214/AJR.06.0827

[16] Berland, L. L., Silverman, S. G., Gore, R. M., Mayo-Smith, W. W., Megibow, A. J., Yee, J., Brink, J. A., Baker, M. E., Federle, M. P., Foley, W. D., Francis, I. R., Herts, B. R., Israel, G. M., Krinsky, G., Platt, J. F., Shuman, W. P., & Taylor, A. J. (2010). Managing incidental findings on abdominal CT: white paper of the ACR incidental findings committee. *Journal of the American College of Radiology : JACR*, *7*(10), 754–773. 10.1016/j.jacr.2010.06.013. 20889105 10.1016/j.jacr.2010.06.013

[17] Mayo-Smith, W. W., Song, J. H., Boland, G. L., Francis, I. R., Israel, G. M., Mazzaglia, P. J., Berland, L. L., & Pandharipande, P. V. (2017). Management of Incidental Adrenal Masses: A White Paper of the ACR Incidental Findings Committee. *Journal of the American College of Radiology : JACR*, *14*(8), 1038–1044. 10.1016/j.jacr.2017.05.001. 28651988 10.1016/j.jacr.2017.05.001

[18] Grumbach, M. M., Biller, B. M., Braunstein, G. D., Campbell, K. K., Carney, J. A., Godley, P. A., Harris, E. L., Lee, J. K., Oertel, Y. C., Posner, M. C., Schlechte, J. A., & Wieand, H. S. (2003). Management of the clinically inapparent adrenal mass ("incidentaloma"). *Annals of internal medicine*, *138*(5), 424–429. 10.7326/0003-4819-138-5-200303040-00013. 12614096 10.7326/0003-4819-138-5-200303040-00013

[19] Zeiger, M. A., Thompson, G. B., Duh, Q. Y., Hamrahian, A. H., Angelos, P., Elaraj, D., Fishman, E., Kharlip, J., American Association of Clinical Endocrinologists, & American Association of Endocrine Surgeons (2009). American Association of Clinical Endocrinologists and American Association of Endocrine Surgeons Medical Guidelines for the Management of Adrenal Incidentalomas: executive summary of recommendations. *Endocrine practice : official journal of the American College of Endocrinology and the American Association of Clinical Endocrinologists*, *15*(5), 450–453. 10.4158/EP.15.5.450. 19632968 10.4158/EP.15.5.450

[20] Young W. F., Jr (2007). Clinical practice. The incidentally discovered adrenal mass. *The New England journal of medicine*, *356*(6), 601–610. 10.1056/NEJMcp065470. 17287480 10.1056/NEJMcp065470

[21] Garrett, R. W., Nepute, J. C., Hayek, M. E., & Albert, S. G. (2016). Adrenal Incidentalomas: Clinical Controversies and Modified Recommendations. *AJR. American journal of roentgenology*, *206*(6), 1170–1178. 10.2214/AJR.15.15475. 27070729 10.2214/AJR.15.15475

[22] Boland, G. W., Dwamena, B. A., Jagtiani Sangwaiya, M., Goehler, A. G., Blake, M. A., Hahn, P. F., Scott, J. A., & Kalra, M. K. (2011). Characterization of adrenal masses by using FDG PET: a systematic review and meta-analysis of diagnostic test performance. *Radiology*, *259*(1), 117–126. 10.1148/radiol.11100569. 21330566 10.1148/radiol.11100569

[23] Sherlock, M., Scarsbrook, A., Abbas, A., Fraser, S., Limumpornpetch, P., Dineen, R., & Stewart, P. M. (2020). Adrenal Incidentaloma. *Endocrine reviews*, *41*(6), 775–820. 10.1210/endrev/bnaa008. 32266384 10.1210/endrev/bnaa008 PMC7431180

[24] Corwin, M. T., Badawy, M., Caoili, E. M., Carney, B. W., Colak, C., Elsayes, K. M., Gerson, R., Klimkowski, S. P., McPhedran, R., Pandya, A., Pouw, M. E., Schieda, N., Song, J. H., & Remer, E. M. (2022). Incidental Adrenal Nodules in Patients Without Known Malignancy: Prevalence of Malignancy and Utility of Washout CT for Characterization-A Multiinstitutional Study. *AJR. American journal of roentgenology*, *219*(5), 804–812. 10.2214/AJR.22.27901. 35731098 10.2214/AJR.22.27901

[25] Thompson L. D. (2002). Pheochromocytoma of the Adrenal gland Scaled Score (PASS) to separate benign from malignant neoplasms: a clinicopathologic and immunophenotypic study of 100 cases. *The American journal of surgical pathology*, *26*(5), 551–566. 10.1097/00000478-200205000-00002. 11979086 10.1097/00000478-200205000-00002

[26] Akbulut, S., Erten, O., Kahramangil, B., Gokceimam, M., Kim, Y. S., Li, P., Remer, E. M., & Berber, E. (2021). A Critical Analysis of Computed Tomography Washout in Lipid-Poor Adrenal Incidentalomas. *Annals of surgical oncology*, *28*(5), 2756–2762. 10.1245/s10434-020-09329-1. 33210268 10.1245/s10434-020-09329-1

[27] Fassnacht, M., Arlt, W., Bancos, I., Dralle, H., Newell-Price, J., Sahdev, A., Tabarin, A., Terzolo, M., Tsagarakis, S., & Dekkers, O. M. (2016). Management of adrenal incidentalomas: European Society of Endocrinology Clinical Practice Guideline in collaboration with the European Network for the Study of Adrenal Tumors. *European journal of endocrinology*, *175*(2), G1–G34. 10.1530/EJE-16-0467. 27390021 10.1530/EJE-16-0467

[28] Choi, Y. A., Kim, C. K., Park, B. K., & Kim, B. (2013). Evaluation of adrenal metastases from renal cell carcinoma and hepatocellular carcinoma: use of delayed contrast-enhanced CT. *Radiology*, *266*(2), 514–520. 10.1148/radiol.12120110. 23151828 10.1148/radiol.12120110

[29] Elhassan, Y. S., Ronchi, C. L., Wijewickrama, P., & Baldeweg, S. E. (2023). Approach to the Patient With Adrenal Hemorrhage. *The Journal of clinical endocrinology and metabolism*, *108*(4), 995–1006. 10.1210/clinem/dgac672. 36404284 10.1210/clinem/dgac672 PMC9999363

[30] Mehmood KT, Sharman T. (2001). Adrenal Hemorrhage. (2001). Treasure Island (FL): StatPearls Publishing; 2022. Available from: https://www.ncbi.nlm.nih.gov/books/NBK555911/. 32310371

[31] Albano, D., Agnello, F., Midiri, F., Pecoraro, G., Bruno, A., Alongi, P., Toia, P., Di Buono, G., Agrusa, A., Sconfienza, L. M., Pardo, S., La Grutta, L., Midiri, M., & Galia, M. (2019). Imaging features of adrenal masses. *Insights into imaging*, *10*(1), 1. 10.1186/s13244-019-0688-8. 30684056 10.1186/s13244-019-0688-8 PMC6349247

[32] Corwin, M. T., Navarro, S. M., Malik, D. G., Loehfelm, T. W., Fananapazir, G., Wilson, M., & Campbell, M. J. (2019). Differences in Growth Rate on CT of Adrenal Adenomas and Malignant Adrenal Nodules. *AJR. American journal of roentgenology*, *213*(3), 632–636. 10.2214/AJR.19.21342. 31039016 10.2214/AJR.19.21342

[33] Pantalone, K. M., Gopan, T., Remer, E. M., Faiman, C., Ioachimescu, A. G., Levin, H. S., Siperstein, A., Berber, E., Shepardson, L. B., Bravo, E. L., & Hamrahian, A. H. (2010). Change in adrenal mass size as a predictor of a malignant tumor. *Endocrine practice : official journal of the American College of Endocrinology and the American Association of Clinical Endocrinologists*, *16*(4), 577–587. 10.4158/EP09351.OR. 20150023 10.4158/EP09351.OR

